# Examining Risk and Protective Factors for Suicidal Behaviors: A Cross-Sectional Study Among Portuguese Male Inmates

**DOI:** 10.3390/healthcare13030293

**Published:** 2025-01-31

**Authors:** Catarina Oliveira, Miguel Basto-Pereira

**Affiliations:** William James Center for Research, Ispa—Instituto Universitário, Rua do Jardim do Tabaco 34, 1149-041 Lisboa, Portugal

**Keywords:** inmates, ideation-to-action, suicidal ideation, suicide attempts, suicidal behaviors

## Abstract

Background/Objectives: Prison inmates face a significantly higher risk of acting on suicidal thoughts than the general population. However, Portuguese studies distinguishing inmates who think about suicide from those who attempt it remain scarce. Given this ideation-to-action distinction, the current cross-sectional study aimed to examine risk and protective factors for suicidal ideation, suicide attempts, and the transition from one outcome to another. Methods: A total of 411 male inmates (M = 37.94 years, SD = 10.91) from 16 Portuguese prisons participated in the study. Data were collected between 2020 and 2024 using four instruments: (1) Sociodemographic Questionnaire; (2) Social and Emotional Competencies Questionnaire (SEC-Q); (3) Predictive Antisocial Spectrum Questionnaire; and (4) Health Risk Behavior Checklist. Results: Among the total sample, 29.1% reported a lifetime history of suicidal ideation, and 19.4% had attempted suicide. Impulsivity/irresponsibility emerged as a key risk factor for suicidal ideation, whereas social and emotional competencies of self-management and motivation were protective factors against suicide attempts. Previous self-harm behavior was the only risk factor for both suicidal ideation and suicide attempts. Conclusions: Our findings provide important insights into risk and protective factors for suicidal behaviors among male inmates, offering key information for policy and practice efforts aimed at reducing mortality in this high-risk population.

## 1. Introduction

Suicidal behaviors are major concerns in public health due to their vastly damaging impact and high prevalence. In fact, suicide is one of the leading causes of death worldwide, accounting for almost 800,000 annual deaths [1]. In Portugal, at least three individuals commit suicide per day, and the vast majority are men [1]. The damaging impact and concerning rates have prompted researchers to understand suicidal behaviors and identify strategies to predict, treat, and prevent suicide. One of the central strategies is the identification of risk factors [2]. The main reviews have shown that suicidal behaviors result from a dynamic and complex interplay among diverse factors. A review by O’Connor and Nock [3] classified risk factors into three categories: (1) personality and individual differences, (2) cognitive and social factors, and (3) negative life events. Within these categories, the main risk factors identified were impulsivity, social isolation, pain tolerance, and a history of trauma.

The main risk factors for suicidal behaviors can also be classified into broader categories. For example, a meta-analysis by Franklin and colleagues [2] identified externalizing and internalizing psychopathology, as well as previous involvement in self-harm behaviors, as main categories. In the externalizing psychopathology category, aggressive and antisocial behaviors, substance abuse, and incarceration history were associated with suicidal ideation and suicide attempts. In the internalizing psychopathology category, the risk of suicidality increased with poor emotion regulation, anxiety disorders, and hopelessness.

In addition to identifying risk factors, it is also crucial to explore protective factors that could mitigate suicidal behaviors. According to Liu and Wang’s review, dimensions like optimism, resilience, future thinking, and goal adjustment can have a protective role [3]. For instance, social and emotional competencies can be considered crucial targets for suicide prevention since they promote better life outcomes [4]. According to the Committee for Children [5], social and emotional competencies can play a significant role in the mitigation of some risk factors of suicidal behaviors. The main protective effects have been found for social and emotional competencies of self-management, relationship skills, responsible decision making, and social/self-awareness [4,5]. For example, both self-management and relationship skills can mitigate the risk impact of hopelessness, anxiety, and substance use. In addition, the impacts of both hopelessness and anxiety can be overcome with the development of self-awareness [5].

Although extant research has been conducted on the protective role of social and emotional skills, evidence regarding factors that protect against suicidal behaviors remains limited [3]. It is particularly crucial to further examine the risk and protective factors of suicidal ideation and suicide attempts since both theoretical and empirical evidence suggests that suicidal ideation and attempts are robust predictors of suicide [6,7]. However, most individuals who consider suicide do not engage in suicidal behaviors. Therefore, suicidal ideation and its transition to attempts may have distinctive risk factors. Based on this distinction between ideation and attempt, a theoretical framework has emerged, called *ideation-to-action* [8,9].

Within this framework, the most comprehensive theory is the Integrated Motivational-Volitional Model of Suicidal Behavior (IMV) [10]. According to the IMV model, the risk of suicidal behaviors begins with specific features within the biosocial context of individuals, including vulnerabilities, privations, and negative life events. The combination of these features can stimulate feelings of defeat and humiliation. If individuals have simultaneous difficulties in overcoming social problems and maladaptive coping styles, these prevailing feelings can give way to perceptions of entrapment. Consequently, the perception of entrapment can increase the risk of suicidal ideation, especially when individuals have poor social support and feel like a burden to others or alienated from their contexts. The last two dimensions are known as perceived burdensomeness and thwarted belongingness, respectively. After the development of suicidal ideation, the risk of suicide attempts is increased by factors like impulsivity and previous suicidal behaviors [10].

Advancing our knowledge about the risk and protective factors of the suicidal process is crucial. However, it is even more urgent for the most vulnerable groups of society. In fact, inmates have a disproportionate risk of considering, attempting, and dying by suicide [11]. According to the World Health Organization [12], suicide is the leading cause of death in prisons, justifying 30% of the mortality rates in these vulnerable contexts. Regarding suicide attempts, the rates for inmates range from 19% to 22%, whereas in the general population, the prevalence is 3% [7]. In addition, the rates of suicidal ideation within inmates range between 34% and 44%, which is particularly concerning given that half of inmates (47–58%) who have suicidal thoughts attempt suicide. The alarming rates of suicidal behaviors in the prison population underscore the need for targeted research and early prevention efforts in prisons [13].

Studies conducted in prisons have suggested that suicidal behaviors in inmates have common risk factors with the general population. In both populations, the main risk factors found are poor emotional regulation, mental health problems [14], and lack of social support [15]. Moreover, in both populations, suicidal behaviors are more prevalent in males [16,17]. In a longitudinal study conducted by Steinhoff and colleagues [18], researchers found that males are at a particularly higher of for not receiving appropriate treatment for self-injury behaviors. Some of these common risk factors seem to be intensified by the prison context, possibly explaining, in part, the higher vulnerability of inmates [8].

The prison context is characterized by restrictive conditions. One of the main restrictions is related to social support, since the prison context can encourage isolation by limiting family and social relationships [19]. In this regard, according to the meta-analysis of Favril and colleagues [7], poor social support is particularly common among inmates who have engaged in suicide attempts. Isolation in prison can be particularly damaging for inmates who have children, since the restrictions on being able to parent can increase the risk of mental health problems characterized by experiences of grief and hopelessness [20]. In this line, Encrenaz and colleagues [21] found that inmates with children were three times more likely to attempt suicide.

Apart from its isolation, the prison context can also restrict involvement in meaningful activities like educational or vocational programs and sports [22]. The prison lifestyle is often sedentary and idle, with monotonous routines [23]. In fact, previous studies conducted in prisons found an association between lack of work activity and both suicidal ideation [6] and suicide attempts [6,24]. In a similar vein, Stoliker [25] found that inmates who engaged in physical exercise were less likely to attempt suicide. These restrictions typically inherent to prisons can lead to hopelessness and anxiety [12]. For most inmates, these feelings of hopelessness and anxiety can stimulate cognitive distortions, where they perceive suicidal behaviors as coping strategies for adaptation, regulation, relief, and temporary avoidance [26].

Understanding the heightened risk of suicidality in prison populations must include an examination not only of prison restrictions, but also of the unique features of inmates, linked to their lifetime and criminal trajectories. Prison populations are already vulnerable before imprisonment [13], possibly because several specific risk factors for suicidality in the general population are also prevalent among inmates and are disproportionately represented within this group. Two factors disproportionately represented in the prison population are high levels of impulsivity and a history of exposure to painful experiences, which means many of these individuals have become accustomed to physical pain, contributing to a reduced fear of death [27]. The reduced fear of death is a dimension known as acquired capability for suicide and is one of the central mechanisms to explain the transition from suicidal ideation to suicide attempts [9,10]. Indeed, inmates have a particularly higher risk of exposure to painful experiences (e.g., violence) given their higher probability for socioeconomic deprivation [6], addiction disorders, and having been exposed to severe adversity during childhood [28]. Similarly, inmates who perpetrate interpersonal violence may have other features that facilitate suicidal behaviors, like higher levels of anger, hostility, and feeling like a burden to others and alienated from their contexts [29].

Taken together, the aforementioned findings suggest that suicidal behaviors in inmates are the result of a dynamic interaction between specific risk factors disproportionately prevalent in their lifetime and criminal trajectories (e.g., impulsivity), as well as factors exclusive to their current context (e.g., prison restrictions) [13]. Exploring this growing evidence base must be an international priority, since several studies have shown that inmates have a higher probability of acting according to their suicidal intentions compared to the general population [30].

Favril and colleagues [8] conducted one of the studies that addressed risk factors of the suicidal process within a prison population. Conducted with a sample of 1203 inmates in Belgium, their findings suggested that a psychiatric diagnosis and self-harm were the main risk factors for suicidal ideation. In addition, inmates who failed to resist their suicidal intentions were those with more involvement in within-prison drug use and self-harm. Furthermore, another study by the same research team suggested that inmates who act upon their suicidal thoughts are more likely to have self-reported diagnoses of mental disorder, self-harm, and substance abuse [30]. In contrast, Stoliker and Abderhalden [31] found that suicidal ideation in inmates was linked with drug use and self-harm, along with poor social support. In addition, the progression of suicidal ideation to attempts was associated with the perpetration of interpersonal violence while intoxicated.

The need for further research within the prison population must also be a national priority, given the alarming prevalence of suicide in Portuguese prisons (11.2% between 2019 and 2020) [32]. Regarding self-mutilation and risk for suicide attempts, in a more recent study conducted by Brandão and colleagues [33], the researchers estimated prevalences of 13% and 21.9% for these health risk behaviors, respectively. To our knowledge, Pragosa [34] conducted the only Portuguese study to address risk factors associated with the transition from suicidal ideation to attempts within a prison context. Conducted in a convenience sample of 104 inmates, the researcher found that inmates who attempted suicide (*n* = 52) were more likely to be single, have a psychiatric diagnosis, and report a history of drug abuse and recidivism. The risk of suicide attempts was also associated with emotional instability, suicidal ideation, and impulsivity. In the interviews conducted by the researcher, the reasons given by inmates for attempting suicide were mainly related to negative affective states, separation from children, and poor social support.

Despite their empirical contributions, some of these international studies did not examine important risk factors like separation from children and impulsivity. Most importantly, none of these international and national studies explored the potential protective role of social and emotional competencies. Despite recognition that social and emotional competencies can mitigate the impact of risk factors of suicidal behaviors in the general population, comparable research is limited regarding prison populations. Building upon these gaps in the literature and addressing the lack of studies focusing on Portuguese prison populations, the present cross-sectional study aimed to investigate key risk and protective factors associated with suicidal behaviors and the suicidal process in inmates. This included factors disproportionately prevalent in their lifetime and criminal trajectories and those potentially linked to the prison context and to painful experiences. In particular, we hypothesize that: (1) social and emotional competencies would be protective factors for suicidal behaviors; and (2) antisocial traits (impulsivity/irresponsibility), having children, lack of physical activities, perpetration of interpersonal violence, drug misuse, and self-harm behaviors would be risk factors for suicidal behaviors. We chose a sample of male inmates, since they represent 93% of the Portuguese inmates [35] and have a higher risk of suicidal behaviors [1,16,17]. To guide our analysis, we adopted central assumptions from ideation-to-action frameworks, aligning with our focus on the underlying mechanisms of suicidality in inmates. Advancing knowledge in this field is particularly important since the findings may help save lives and promote safer prison environments [15].

## 2. Materials and Methods

### 2.1. Study Design and Participants

The current study adopts a cross-sectional design. The data were collected between 2020 and 2024. The total sample consisted of 411 male inmates. Participants were selected from a total of 16 prisons in central and northern Portugal. Among the total sample, the mean age was 37.94 years (SD = 10.91, range: 19–83). On average, they had 8.69 (SD = 3.02) years of education. Most were from the ethnic majority group (78.6%, *n* = 316) and were single (63.7%, *n* = 261). Within this total sample, our main interest was in the participants with suicidal ideation (29.1%; *n* = 119). The mean age of this subgroup was 39.3 years (SD = 10.55, range: 20–67). On average, they had 8.31 (SD = 3.22) years of education and were from the ethnic majority group (75%, *n* = 87).

### 2.2. Instruments

#### 2.2.1. Sociodemographic Questionnaire

This questionnaire assessed sociodemographic data on gender, age, nationality, ethnic or cultural minority status (defined as participants who reported and perceived themselves as having racial or ethnic roots different from those of the majority Portuguese population), educational attainment, and marital status. We also used this questionnaire to identify potential risk factors relevant to the prison context, such as separation from children and lack of physical activity (based on regular sports participation). In both cases, these variables were operationalized using dichotomous (yes/no) questions.

#### 2.2.2. Social and Emotional Competencies Questionnaire (SEC-Q)

The multidimensional SEC-Q self-report measure assesses 16 items grouped into four dimensions: (a) self-awareness (e.g., “I know how to label my emotions”), which represents the ability to label emotions and recognize individual strengths; (b) self-management and motivation (e.g., “I can motivate myself”), which involves the management of one’s own emotions to pursue goals; (c) social awareness and pro-social behavior (e.g., “I pay attention to the needs of others”), which includes social competencies like perspective taking and the ability to relate to others; and (d) decision making (e.g., “I don’t make decisions carelessly”), which is the ability to analyze situations while respecting others [36,37]. Participants rate their level of agreement with each item within the last 12 months using a 5-point Likert scale (1 = *Strongly disagree* to 5 = *Strongly agree*). Higher scores in each dimension represent better skills within that specific dimension [37]. The original [37] and Portuguese [36] versions of the SEC-Q showed good psychometric properties. In the original version [37], the authors found good reliability of the dimensions in young adults (α = 0.77–0.83), as well as divergent and concurrent validity. The findings suggested negative significant correlations with difficulties in identifying (*p* = −0.04–−0.38) and expressing feelings (*p* = −0.08–−0.42), as well as positive significant correlations with total perceived emotional intelligence (*p* = 0.21–0.46). In the Portuguese [36] version, the authors found satisfactory levels of internal consistency (α = 0.71–0.79), as well as concurrent (*p* = 0.23–0.34) and divergent validity (*r* = −0.11–−0.15). In the current study, this instrument showed appropriate internal consistency (α = 0.75–0.80).

#### 2.2.3. Predictive Antisocial Spectrum Questionnaire

This self-report instrument assesses antisocial traits in forensic populations with 10 items divided into two dimensions: (a) impulsivity/irresponsibility (e.g., “If I need to take risks, I take them, even if it affects my safety”), which reflects both disregard for consequences and acting without thinking, and (b) interpersonal relationships (e.g., “I have already gotten into trouble for risking my own safety or others”), which represents the individual’s ability to relate to others [38]. Participants rate the degree to which each item describes them on a 4-point Likert scale (1 = *Strongly disagree* to 4 = *Strongly agree*) from the beginning of their adolescence (before 15 years old). The Predictive Antisocial Spectrum questionnaire was developed in Portugal, and the 10-item version has shown good psychometric proprieties [38]. The author found an appropriate reliability for the dimensions (α = 0.68–0.81) and a positive moderate concurrent validity (*r* = 0.36–0.52). In the current study, this instrument also demonstrated appropriate internal consistency (α = 0.69–0.77).

#### 2.2.4. Health Risk Behavior Checklist

This checklist is a self-report measure that assesses 15 health risk behaviors (early smoking, sexual intercourse, alcohol and drug initiation, sedentary lifestyle, alcohol abuse, drug misuse, perpetration of interpersonal violence, self-harm behaviors, suicidal ideation, suicide attempts, inadequate meals, carrying weapons, not brushing teeth, sexual intercourse without condom) with 20 items. Some of the items are scored with a 5-point Likert scale (1 = *Very often* to 5 = *Never*), while others are scored as dichotomous responses (*Yes* or *No*). A risky behavior is considered present with an affirmative response on the yes/no items or a response of *sometimes*, *often*, or *very often* on the Likert scale items. For the risk factors early smoking, alcohol and drug initiation, and early sexual intercourse, the presence is considered if the participants had these experiences at an age younger than 16 years. The timing of these health risk behaviors includes current (for early smoking, sexual intercourse, alcohol and drug initiation, sedentary lifestyle, alcohol abuse, drug misuse, inadequate meals, not brushing teeth), lifetime (for perpetration of interpersonal violence, self-harm behaviors, suicidal ideation, suicide attempts), last week (for inadequate meals), last 30 days (for carrying weapons), and last time (for sexual intercourse without condom). The instrument provides a global index of health risk behaviors by summing the number of risk factors identified. The global score ranges from 0 to 20 [39].

In the present study, this instrument was employed to gather data on suicidal behaviors and theoretical related risk factors (e.g., [10]) such as drug misuse, previous involvement in self-harm behaviors, and perpetration of interpersonal violence. Drug misuse included prior drug experimentation (e.g., marijuana, cocaine, heroin, methamphetamine or ecstasy) and the present consumption. Previous involvement in self-harm behaviors involved acts of intentional self-harm (e.g., make scratches or cuts in parts of the body with a sharp object, burn the skin with the end of a cigarette or a lit match). Perpetration of interpersonal violence comprised the involvement in previous violent behaviors, such as fights. All these variables were scored as dichotomous responses (*yes* or *no*). The outcomes were measured using single-item measures (“Have you ever seriously thought about taking your own life?” and “If so, have you ever attempted suicide?”, respectively) scored as dichotomous responses (*yes* or *no*). The lifetime assessment of suicidality allows for an evaluation of suicide risk across the criminal trajectories of men who ultimately become incarcerated, which is consistent with recent studies on prison populations [31]. This checklist, developed for Portugal, has demonstrated appropriate psychometric properties and is part of the official base of psychological assessment instruments of the American Psychological Association (APA) [39].

### 2.3. Ethics

This study was part of the research project *Assessment for effective interventions: Reducing the risk of criminal recidivism and social marginalization*. The aim of this project was to analyze the risk and protective factors of both pro/antisocial behavior and social integration in adults, in both communities and prison populations. Ethical approval for the study protocol was granted by the Ispa Ethics Committee (I/029/01/2020).

Prior to the administration of the questionnaires, team members who supervised the data collection explained to participants the aims of the project, the confidential and voluntary nature of participation, as well as the participants’ right to withdraw at any time. There were no financial incentives for participation. The participants who agreed to engage in the study gave written informed consent, which was then separated from the questionnaires to guarantee confidentiality.

### 2.4. Procedure

After obtaining approval from the Ethics Committee, authorizations were requested from the Directorate-General for Social Reintegration and Prison Services (DGRSP) and from the directors of each prison where the research team conducted the study.

The data collection process was coordinated with each prison director via email and/or telephone. The exclusion criteria included all conditions that could have an influence on participants’ ability to consent to the study or to understand and respond to the questionnaires (e.g., illiteracy, not understanding Portuguese, and having an intellectual disability or severe psychopathology that made it impossible to respond to the questionnaires). Participants on remand were also excluded. The sample was recruited from 16 Portuguese prisons of central and north Portugal. The prisons were selected according to their geographical proximity and the number of participants that met the criteria. Prison staff were asked to randomly select participants from the complete list of inmates at the facility.

The paper-and-pencil Portuguese questionnaires were administered to a group of six to ten inmates at a time. The administration was supervised by two to four research team members in spaces inside the prisons (e.g., library, school, visiting rooms). On average, the participants completed the protocols in approximately 25 min. If the researcher was unable to contact a participant (e.g., due to hospitalization), the participant was replaced by another inmate drawn from the premade random list.

### 2.5. Analytic Strategy

Data analysis was conducted using IBM SPSS (Version 29). First, bivariate analyses (unadjusted ORs) were performed to examine independent associations between suicidal indicators and sociodemographic characteristics (age, education, and minority status), as well as theoretically relevant dimensions identified as protective factors (social and emotional competencies) and risk factors (e.g., drug misuse and self-harm behaviors) for suicidal ideation, suicide attempts, and the suicidal process (i.e., factors increasing the likelihood of suicide attempts among inmates self-reporting suicidal ideation). Second, binary logistic regression was employed to include variables with significant associations identified in the bivariate analysis. This approach enabled the calculation of adjusted odds ratios (ORs) while controlling for significant protective factors, risk factors, and sociodemographic characteristics. This methodological strategy reduced the risk of underpowered analyses and improved the robustness of the results. All assumptions for conducting binary logistic regression were assessed and satisfied, with the exception of the self-awareness subscale, which was excluded from the study. Additionally, cases with missing data (*n* = 8) were removed from the analysis.

## 3. Results

### 3.1. Prevalence Estimates

As shown in Table 1, among the 411 inmates, 119 participants reported having lifetime suicidal ideation (29.1%). The remaining participants did not report having suicidal ideation (70.6%; *n* = 290), and two cases had missing values. Among the total sample, 79 participants had attempted suicide (19.4%). All participants who reported engaging in suicide attempts simultaneously reported suicidal ideation. Most of the 411 participants did not report suicide attempts (80.6%; *n* = 329), and three cases had missing values. Further prevalences and descriptive statistics of the total sample and subsamples are listed in Table 1.

### 3.2. Bivariate Analyses

Table 2 shows the results of the bivariate analyses. Antisocial traits, impulsivity/irresponsibility, and previous involvement in self-harm behaviors increased the odds of both suicidal ideation and suicide attempts. Self-management and motivation was the only social and emotional dimension decreasing the odds of both suicidal ideation (OR = 0.72; *p* = 0.03) and suicide attempts (OR = 0.62; *p* < 0.001). Among the inmates with suicidal ideation, previous involvement in self-harm behaviors (OR = 4.10; *p* = 0.002) increased the odds of suicide attempts.

### 3.3. Multivariate Analyses

The model obtained for suicidal ideation was statistically significant (X^2^ (8) = 59.31, *p* < 0.001). The model explained 21% (Nagelkerke R^2^) of the variance in suicidal ideation and correctly classified 77% of the cases. In the multivariate model, antisocial traits–impulsivity/irresponsibility (OR = 1.44; 95% CI: 1.01–1.04) and previous involvement in self-harm behaviors (OR = 5.59; 95% CI: 3.14–9.96) increased the odds of suicidal ideation after adjusting for sociodemographic variables (see Table 3). The model obtained for suicide attempts was also statistically significant (X^2^ (8) = 67.65, *p* < 0.001). The model explained 27% (Nagelkerke R^2^) of the variance in suicide attempts and correctly classified 85% of the cases. Previous involvement in self-harm behaviors predicted suicide attempts (OR = 8.57%; 95% CI: 4.58–16.03), and the social and emotional competency of self-management and motivation (OR = 0.59; 95% CI: 0.41–0.87) was negatively related to suicide attempts (see Table 3).

The logistic regression model obtained for the transition was also statistically significant (X^2^ (4) = 9.18, *p* = 0.05). The model explained 11% (Nagelkerke R^2^) of the variance in the transition and correctly classified 67.6% of the cases. Among the subsample of inmates with suicidal ideation, only previous involvement in self-harm behaviors predicted the transition to suicide attempts (OR = 3.71; 95% CI: 1.47–9.35) (see Table 3).

## 4. Discussion

In Portugal, this study is among the few that have explored the risk factors for suicidal ideation and its transition to suicide attempts among inmates. Consistent with previous research, our findings suggest that incarcerated individuals in Portugal represent a high-risk group for suicide [12]. In fact, more than half of the participants who reported suicidal ideation had already attempted suicide. This study indicates that Portuguese male inmates are at an even higher risk of suicide compared to the prevalence reported in other countries [8,30], emphasizing the urgent need for further investigation as well as early prevention and intervention efforts within Portuguese prison settings.

Our study contributes to the literature with three main findings. First, as expected, previous involvement in self-harm behaviors predicted suicidal ideation and the transition to suicide attempts. Our findings are consistent with previous studies that have found associations between self-harm behaviors, suicidal ideation [8,31], and suicide attempts [30]. Additionally, contrary to what was expected, impulsivity/irresponsibility was the only risk factor for suicidal ideation, which is inconsistent with previous studies [34]. Finally, self-management and motivation was the only protective factor against suicide attempts. Indeed, previous studies have identified some dimensions related to competencies of self-management and motivation (e.g., goal adjustment, problem solving) as key protective factors against suicide attempts [3]. Taken together, our findings suggest that suicidal behaviors may share common risk factors (e.g., self-harm behaviors), despite differences in protective factors.

According to ideation-to-action theories, such as the Integrated Motivational–Volitional Model of Suicidal Behavior (IMV) [10], some of the central risk factors for the transition from suicidal ideation to suicide attempts are related to painful experiences due to a lower pain sensitivity and a diminished fear of death over time [9,10,27]. Lower pain sensitivity and the fear of death can be stimulated by self-harm [40], explaining, therefore, the role of self-harm in the transition from suicide ideation to suicide attempts. Also, impulsivity is seen as a promoter of the transition to suicide attempts [10], given its association with poor premeditation [9]. Hence, impulsivity can hinder resistance to suicidal ideation [30]. However, in our study, impulsivity predicted suicidal ideation instead of the transition to suicide attempts. Our findings may suggest that inmates with suicidal ideation might be at an increased risk of attempting suicide, which may also explain the association of suicidal ideation with self-harm behaviors. Indeed, suicidal ideation can be developed across a spectrum of intensity that starts with the general intent to die and ends with a plan with details and the intention to achieve it [41]. Hence, it is possible that the inmates with suicidal ideation are already at the end of this spectrum, which is concerning, particularly because previous studies have shown [30] that inmates have twice the probability of acting according to suicidal thoughts compared to the general population.

Despite identifying risk factors of suicidal behaviors, this study also examined social and emotional competencies as protective factors against suicidal behaviors. Our findings suggest that the social and emotional competencies of self-management and motivation are protective factors for suicide attempts. Self-management and motivation involve the regulation of one’s own emotions to pursue goals [36,37]. Therefore, our findings support previous research that has demonstrated poor emotion regulation as a main risk factor of suicidal behaviors [2,14], as well as goal adjustment as a protective factor against suicide attempts [3]. Our findings are also consistent with other ideation-to-action theories, such as the Three-Step-Theory [9], which conceptualizes difficulties in emotion regulation as one the central risk factors for suicidal behaviors.

Surprisingly, in the current study, separation from children and the perpetration of interpersonal violence among male inmates did not appear to predict suicidal ideation and suicide attempts. Regarding having children, in previous research, this factor was associated with suicide attempts [21]. However, previous studies also found that inmates with children tend to have more education along with convictions not related to violent crimes [42]. Furthermore, many inmates with children tend to be resilient and persistent in maintaining their parental role [20]. Regarding the perpetration of interpersonal violence, the inconsistency in our results can potentially be explained by methodological differences from previous studies. For instance, in Stoliker and Abderhalden [31], the perpetration of interpersonal violence was considered while inmates were intoxicated. In addition, inmates who perpetrate interpersonal violence may have other characteristics that facilitate suicidal behaviors, for example, feeling like a burden to others and alienated from their contexts [29].

To our knowledge, this is the first study in Portugal to simultaneously examine both risk and protective factors against suicidal behaviors and suicidal processes among a large sample of incarcerated males. In fact, few studies worldwide have simultaneously examined the impacts of both risk and protective factors on suicidal behaviors among incarcerated individuals. Despite the important contributions, our findings must be interpreted considering relevant limitations. First, given the cross-sectional nature of our research design, we were unable to examine causal relationships [43] or provide an accurate examination of the suicidal process across time. Another methodological limitation is the retrospective self-report nature of our research design, which can introduce biases, such as those related to social desirability [44]. Finally, we were unable to examine the prevalence of suicidal ideation and attempt among inmates after imprisonment, which hinders our understanding of the exclusive impact on the prison context. Each of these limitations suggests key lines of future research.

### Practical Implications

Despite its limitations, our study contributes to the existing literature and offers significant practical implications. We have addressed one of the most concerning mental health issues, with the most damaging outcome, in one of the most high-risk populations. Our findings highlight the importance of implementing psychosocial programs responsive to the unique needs of inmates. In line with our findings, these programs must target the development or improvement of the protective factors found, including social and emotional competencies. Additionally, these programs must address the warning indicators of suicidal behaviors, such as impulsivity and a history of prior or current involvement in self-harm behaviors. In addition to healthcare services, the prison staff should receive appropriate training to better identify these indicators.

In this study, the obvious warning indicator factor was suicidal ideation. All inmates with suicide attempts reported prior suicidal thoughts. Therefore, the prevention of suicidal behaviors in prisons should also target the early stages of the suicidal process to identify inmates at risk of development of suicidal ideation [15]. For this purpose, initial health screening evaluation must be implemented. Such an approach allows for a risk and needs assessment for each inmate. Subsequently, the inmates with suicidal ideation must be monitored and provided with continuous and appropriate access to healthcare services [12]. Ensuring proper health services in prisons is a necessary step in building a healthier and safer society [45].

Therefore, improving healthcare services and providing key information for prevention and intervention strategies could help to prevent progression through the suicidal process before it reaches its worst and irreversible end—suicide.

## 5. Conclusions

In summary, this cross-sectional work adds to the literature by identifying potential risk and protective factors of suicidal behaviors in Portuguese male inmates. Our findings are in line with the hypothesis that self-harm behaviors predict suicidal ideation and suicide attempts. Our study suggests that, while impulsivity appears to be pivotal in contributing to suicidal ideation, some social and emotional competencies may work as protective factors against suicide attempts.

Additionally, our work points to pivotal directions for future studies. Future studies should adopt research designs with higher methodological quality (e.g., long-term longitudinal studies), allowing for better identification and understanding of the risk and protective factors of the suicidal process across time. In addition, in prison populations, suicidal ideation seems to be more prevalent in older inmates [46]. For instance, examining the impact of age on suicidal behaviors could be an important research focus.

Another important avenue for future research may involve addressing suicidal risk among female inmates and inmates on remand, as some studies suggest that female inmates are more likely to attempt suicide [17] and that inmates on remand have a higher risk of suicide [11]. Finally, to better understand the impact of the prison context, future studies might examine changes in the prevalence of suicidal ideation and attempts among inmates before and after imprisonment. These future directions may enhance our understanding of the risk and protective factors for suicidal behaviors, providing key information for policy and practice efforts aimed at reducing mortality in this high-risk population.

## Figures and Tables

**Table 1:** Sociodemographic characteristics and descriptive statistics.

Variables			Male Inmates (*N* = 411)			Male Inmates with SI (29.1%; *n* = 119)			Male Inmates with SA (19.4%; *n* = 79)		
			M	SD	MV	M	SD	MV	M	SD	MV
Sociodemographic factors	Age		37.94	10.91	3	39.24	10.55	1	38.5	10.18	1
	Education		8.69	3.01	7	8.31	3.22	4	8.32	3.03	2
			n	%	MV	n	%	MV	n	%	MV
	Minority Group		86	21.4%	9	29	25%	3	20	26%	2
	Marital Status	Single	261	63.7%	1	74	62.2%	0	51	64.6%	0
		Relationship similar to marital status	56	13.7%		17	14.3%		10	12.7%	
		Married	37	9%		6	5%		3	3.8%	
		Divorced/Separated	55	13.4%		22	18.5%		15	19%	
		Widower	1	0.2%		0	0%		0	0%	
Quantitative Protective factors—Social and Emotional Competencies	Self-management and motivation		3.46	0.70	7	3.32	0.82	4	3.24	0.83	4
	Social awareness and pro-social behaviors		3.21	0.62	8	3.13	0.62	2	3.19	0.56	2
	Decision making		3.02	0.85	2	2.98	0.90	1	2.95	0.92	1
Quantitative Risk factors—Antisocial Spectrum	Impulsivity/irresponsibility		0.97	0.77	10	1.13	0.78	4	1.16	0.79	3
	Interpersonal relationships		1.53	0.85	15	1.52	0.86	6	1.54	0.85	4
			n	%	MV	n	%	MV	n	%	MV
Dichotomous risk factors	Having children		268	65.2%	0	78	65.5%	0	52	65.8%	0
	Non-participation in sports		125	30.6%	3	39	33.3%	2	25	32.1%	1
	Perpetration of interpersonal violence		266	64.7%	0	76	63.9%	0	48	60.8%	0
	Drug misuse		286	69.6%	0	78	65.5%	0	51	64.6%	0
	Self-harm behaviors		76	18.5%	0	48	40.3%	0	40	50.6%	0

Abbreviations: M, mean; SD, standard deviation; MV, missing values; n, total of participants; %, percentage of participants; SI, inmates with suicidal ideation; SA, inmates with SI and suicide attempts.

**Table 2:** Unadjusted odds ratio between sociodemographic factors, risk and protective factors, and suicide outcomes.

*Variables*	Suicidal Ideation (Ref. = No)				Suicide Attempts (Ref. = No)				Transition (Ref. = No)			
	OR	SE	95% CI	*p*	OR	SE	95% CI	*p*	OR	SE	95% CI	*p*
Sociodemographic factors												
Age	1.01	0.01	0.99–1.03	0.16	1.01	0.01	0.98–1.03	0.61	0.98	0.02	0.95–1.02	0.29
Education	0.95	0.04	0.88–1.02	0.14	0.95	0.04	0.87–1.04	0.24	1.00	0.06	0.89–1.13	0.96
Minority group (Ref. = No)	1.22	0.27	0.73–2.06	0.45	1.38	0.29	0.67–2.45	0.28	1.17	0.46	0.47–2.88	0.63
Protective factors—social and emotional Competencies dimensions												
Self-management and motivation	0.72	0.15	0.54–0.97	0.03	0.62	0.16	0.45–0.86	<0.01	0.71	0.27	0.42–1.20	0.20
Social awareness and pro-social behaviors	0.74	0.18	0.53–1.04	0.09	0.95	0.20	0.64–0.41	0.79	1.60	0.32	0.86–1.97	0.14
Decision making	0.93	0.17	0.72–1.20	0.56	0.89	0.15	0.67–1.19	0.43	0.89	0.22	0.58–1.38	0.61
Quantitative risk factors—antisocial spectrum dimensions												
Impulsivity/irresponsibility	1.45	0.14	1.09–1.92	<0.01	1.49	0.16	1.09–2.04	<0.01	1.20	0.26	0.72–1.98	0.49
Interpersonal relationships	1.00	0.13	0.78–1.30	0.96	1.01	0.15	0.75–1.36	0.94	1.06	0.23	0.67–1.67	0.80
Dichotomous risk factors												
Having children (Ref. = No)	1.00	0.23	0.64–1.57	0.99	1.03	0.26	0.62–1.73	0.90	1.04	0.41	0.47–2.31	0.93
Non-participation in sports (Ref. = No)	0.87	0.24	0.55–1.39	0.57	0.92	0.27	0.54–1.57	0.76	1.19	0.41	0.53–2.67	0.68
Perpetration of interpersonal violence (Ref. = No)	1.03	0.23	0.66–1.62	0.90	0.81	0.26	0.49–1.34	0.41	0.66	0.42	0.29–1.50	0.32
Drug misuse (Ref. = No)	0.76	0.23	0.48–1.20	0.23	0.75	0.26	0.45–1.26	0.28	0.88	0.41	0.39–1.96	0.75
Self-harm behaviors (Ref. = No)	5.50	0.27	3.24–9.32	<0.01	8.43	0.29	4.81–14.77	<0.01	4.10	0.46	1.68–10.01	<0.01

Abbreviations: OR, adjusted odds ratio; SE, standard error; 95% CI, confidence interval; Ref., reference category.

**Table 3:** Binary logistic regression for suicidal outcomes.

	Suicide Outcomes											
	Suicidal Ideation (Ref. = No)				Suicide Attempts (Ref. = No)				Transition (Ref. = No)			
Type of Factors	*β*	*OR*	*SE*	*p*	*β*	*OR*	*SE*	*p*	*β*	*OR*	*SE*	*p*
Sociodemographic factors												
Age	0.03	1.03	0.01	<0.01	0.03	1.03	0.02	0.06	0.002	1.00	0.02	0.91
Education	−0.04	0.96	0.04	0.40	−9−0.02	0.98	0.05	0.76	0.04	1.04	0.07	0.54
Minority Group (Ref. = No)	0.16	1.18	0.30	0.59	0.23	1.26	0.05	0.51	0.21	1.23	0.50	0.68
Protective factors	-	-	-	-	-	-	-	-	-	-	-	-
Self-management and motivation	−0.33	0.72	0.18	0.06	−9−0.52	0.59	0.20	<0.01	-	-	-	-
Risk factors												
Self-harm behaviors (Ref. = No)	1.72	5.59	0.29	<0.01	2.15	8.57	0.32	<0.01	1.31	3.71	0.47	<0.01
Impulsivity/irresponsibility	0.36	1.44	0.18	0.04	0.23	1.26	0.21	0.27	-	-	-	-
Models												
X^2^ (df), *p*	59.31 (8), *p* < 0.001				67.65 (8), *p* < 0.001				9.18 (4), *p* = 0.05			
Nagelkerke R^2^	0.21				0.27				0.11			
%	77.1%				85%				67.6%			

Abbreviations: OR, adjusted odds ratio; SE, standard error; 95% C.I, confidence interval X^2^, chi square; df, degrees of freedom; %, percentage of correct classification; Ref., reference category.

## Data Availability

The datasets presented in this article are not readily available because the data are part of an ongoing study. Requests to access the datasets should be directed to the corresponding author.
